# Print Media Role and Its Impact on Public Health: A Narrative Review

**DOI:** 10.7759/cureus.59574

**Published:** 2024-05-03

**Authors:** Sushim Kanchan, Abhay Gaidhane

**Affiliations:** 1 School of Epidemiology and Public Health, Jawaharlal Nehru Medical College, Datta Meghe Institute of Medical Sciences, Wardha, IND

**Keywords:** health education, health behavior change, health information dissemination, health literacy, health information, digital media, print media

## Abstract

Print media plays a pivotal role in communicating public health information, acting as a vital channel for spreading awareness and encouraging healthy behaviors. This narrative review delves into the historical significance of print media in health communication while evaluating its relevance in today's digital media environment. Despite encountering significant hurdles like digital competition and financial limitations, print media remains indispensable for addressing crucial health issues and disseminating information during public health emergencies. Effectively utilizing print media for health promotion necessitates careful planning, thorough evaluation, and targeted distribution to ensure widespread impact and audience engagement. Employing a comprehensive search strategy, relevant literature was identified through electronic databases and manual searches of reference lists. The gathered literature underwent rigorous screening to align with the review's objectives, with key insights synthesized through iterative analysis. Print media remains a cornerstone of health communication, offering tangible avenues for information dissemination and audience interaction. However, its efficacy is subject to various factors, including technological advancements, evolving media landscapes, and challenges in content dissemination. Overcoming these obstacles requires innovative approaches and collaborative endeavors to harness the full potential of print media in advancing public health objectives.

## Introduction and background

Media plays a pivotal role in disseminating information to the public and holds significant importance in raising awareness about various issues. It serves as a potent tool for creating societal consciousness. Print media, encompassing newspapers, magazines, brochures, and posters, serves as a traditional yet enduring medium for conveying health-related messages. Newspapers, in particular, have been instrumental in providing information about diseases and outlining measures implemented by central and state governments to curb infections and enforce lockdowns [1]. In 1998, research conducted by the American “National Health Council” revealed that 75% of individuals obtain health-related news through the media, with 40% relying on television, 35% on magazines or journals, 16% on newspapers, and 2% through the Internet [2]. The extensive coverage in newspapers has proven effective in addressing gaps and offering guidance to the public during critical times, fostering hygienic practices. Even in the current digital era, print media continues to play a robust role in generating awareness. Recognizing the importance of health communication and the historical significance of print media in this context forms the foundation for this narrative review.

Objectives of the review

In framing this narrative review, the overarching objectives are twofold, aiming to elucidate and critically examine the role of print media in public health communication. Firstly, this review seeks to provide a comprehensive overview of the historical and contemporary importance of print media, highlighting its contributions to health education, information dissemination, and awareness campaigns. Secondly, the review aims to assess the effectiveness of print media in influencing public health outcomes. By evaluating the strengths, challenges, and opportunities associated with print media, the goal is to offer insights that can inform future health communication strategies and practices. Through a nuanced exploration of the role and impact of print media, this review strives to contribute to the ongoing dialogue surrounding effective approaches to health communication in diverse and dynamic societal contexts. Our review focuses on print media's historical and existing significance, putting light on its role in health education, information distribution, and awareness programs. Additionally, the review evaluates the effectiveness and impact of print media in shaping human health behavior.

## Review

Methodology

**Figure 1:**
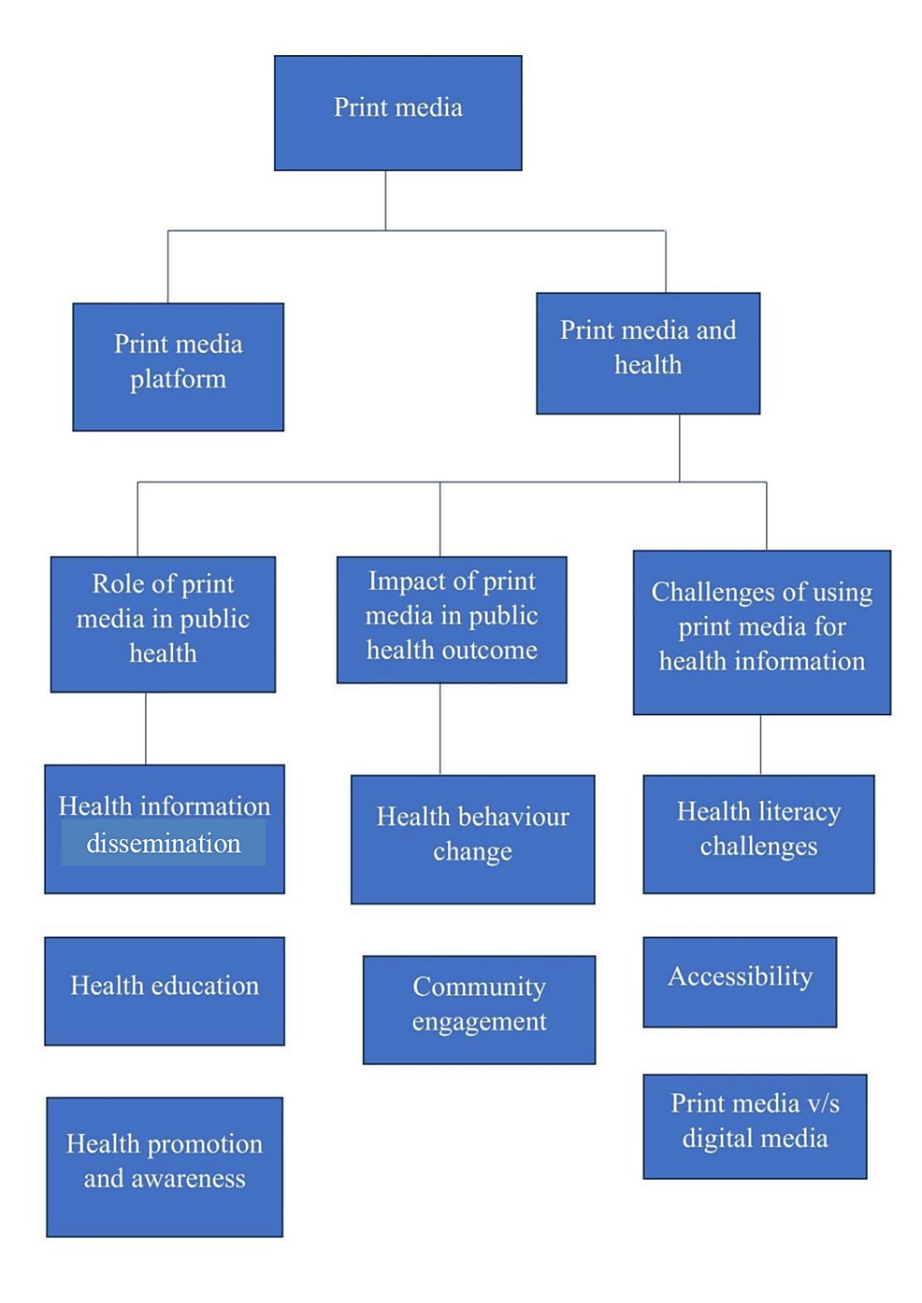
Summary of the study

**Figure 2:**
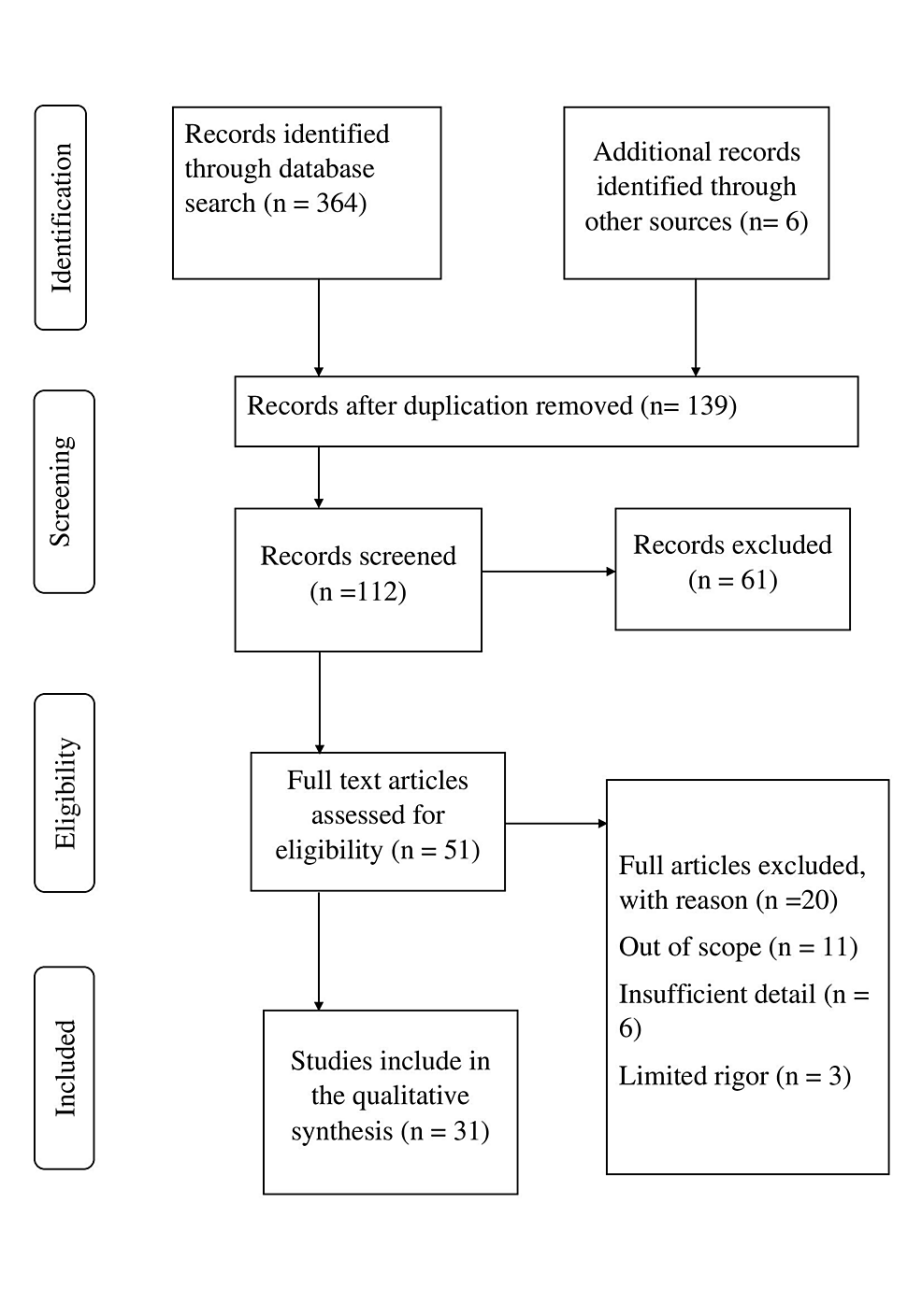
Inclusion and exclusion criteria of the study

This narrative review synthesizes existing literature to examine the role of print media in public health communication. A comprehensive search strategy was employed to identify relevant studies, including peer-reviewed articles, reports, and academic publications, from electronic databases such as PubMed, Google Scholar, and Scopus. Keywords and phrases related to print media, health communication, public health, health literacy, and behavior change were used to retrieve relevant literature. The search was not limited by publication date, and studies published up to the present were included to ensure a comprehensive overview of the topic. Additionally, reference lists of relevant articles were manually reviewed to identify additional sources that met the inclusion criteria. The retrieved literature was screened based on relevance to the objectives of the review, which focused on exploring the historical and contemporary significance of print media in health communication, assessing its effectiveness in influencing public health outcomes, and identifying challenges and opportunities associated with its utilization. Our research question, "How has print media historically contributed to public health communication, and what are its current implications for promoting health awareness, disseminating information, and influencing behavior change?" guided the structure of the review. Beginning with an exploration of "Print Media Platforms," various forms of print media used in public health communication were examined, followed by an in-depth analysis of the "Role of Print Media in Public Health," encompassing its involvement in health information dissemination, education, and promotion. Subsequently, the focus shifted to the "Impact of Print Media in Public Health Outcome," highlighting its role in driving behavior change and fostering community engagement. Addressing the "Challenges of Using Print Media for Health Information" concluded the discussion before synthesizing key findings and implications for future research and practice. Limitations of the methodology include potential bias in the selection of literature and the exclusion of non-English language publications. However, efforts were made to minimize bias by employing a systematic search strategy and transparent inclusion criteria. Overall, this methodology facilitated a rigorous and systematic review of the literature on print media in public health communication, providing valuable insights into its role, effectiveness, and challenges in promoting health education, disseminating information, and shaping public perceptions. Figure 1 shows the summary status of the article, and Figure 2 depicts the inclusion and exclusion criteria.

Print media platforms

Despite facing fierce competition from newer forms of media, such as the Internet and television, print media continues to hold relevance in certain contexts. One of the most prominent forms of print media is newspapers, which historically were published in various formats, including national broadsheets, state-wide editions, and local publications, often on a daily or weekly basis. Newspapers typically cover a wide range of topics, including national and international news, local events, sports, weather updates, opinion pieces, business news, and crime reports. Additionally, newspapers may feature comic strips, puzzles, or advice columns to cater to diverse reader interests.

Magazines, another form of print media, distinguish themselves from newspapers by their emphasis on visual elements, with more graphics and fewer articles. Magazines often target specific niche audiences and frequently include glossy advertisements tailored to their demographic [3]. Books, a cornerstone of print media, have played a crucial role in both entertainment and education for centuries [3]. The invention of the printing press revolutionized book production, making books more affordable and accessible to the general public. Books encompass a wide range of genres and categories, including textbooks for education, encyclopedias for knowledge storage, and novels for entertainment [4]. Billboards, often seen along highways and busy shopping areas, are another form of print media used for advertising [5]. While traditional print billboards remain effective at capturing attention, electronic billboards are becoming increasingly popular due to their ability to display dynamic, attention-grabbing content [3]. Posters serve a similar purpose to billboards but are typically used in more localized settings to advertise products or events [4]. One of the advantages of posters is their cost-effectiveness compared to other forms of advertising media such as television or radio ads. Other print media platforms include flyers, coupons, zines, yard signs, periodicals, comic books, postcards, greeting cards, letters, and printed photographs, each serving unique purposes in communication and marketing strategies [4]. Despite the rise of digital media, print media continues to offer tangible and effective ways to disseminate information and engage with audiences in various contexts.

Role of print media in public health

The newspapers have been diligently providing comprehensive information about diseases and the measures implemented by both central and state governments to curb the spread of infections and enforce lockdowns. This extensive coverage in newspapers has not only facilitated governments in identifying and addressing loopholes but has also served as a conduit for offering suggestions to the general populace during this critical period [2]. Furthermore, print media has been instrumental in promoting hygienic habits among the population. Indeed, media, particularly print media, has historically played a crucial role in disseminating vital information during public health crises. Notably, print media has been instrumental in spreading awareness during significant health initiatives such as the Polio Eradication Mission, Tuberculosis, AIDS, Encephalitis, H1N1 Swine Flu, and various other diseases [2]. Special stories and prominently placed news articles related to these health concerns have graced the front pages of newspapers, ensuring widespread awareness and encouraging precautionary measures. A study conducted by the American “National Health Council” in 1998 highlighted the significant role of media in health communication, with 75% of people relying on media outlets for health-related news, with TV accounting for 40%, magazines or journals for 35%, newspapers for 16%, and the internet for 2% [2].

Dissemination of Health Information

A mass media approach, including printed brochures, has long been a staple in health communication strategies. Print media, comprising newspapers, magazines, brochures, posters, and other tangible formats, has consistently served as a crucial vehicle for disseminating health information [6]. This traditional yet enduring medium plays a pivotal role in educating and empowering diverse populations. From conveying preventive measures and disease awareness to promoting healthy behaviors, print media has been instrumental in delivering a wide array of health-related messages. Its tangible nature and wide reach make print media a versatile tool for public health communication. As technological advancements continue to reshape the media landscape, understanding the historical and contemporary significance of print media in health dissemination becomes imperative for maximizing its role in fostering informed communities and positively impacting public health outcomes. Over the past three decades, numerous studies have explored how news is sought and shaped by journalists within media organizations, while others have proposed strategies for increasing news coverage of significant health and medical issues [7]. In South Africa, the reliance on printed media for information dissemination to the public is deeply ingrained in the communication practices of most government departments and institutions [8]. Printed brochures are prominently utilized to provide information about conditions such as schizophrenia to patients and caregivers [7].

A study by Redmond et al. [9] delves into the effectiveness of health information sources among different socioeconomic and demographic groups. It investigates whether interpersonal sources (friends, family, community organizations, healthcare providers) or mass media sources (print, TV, Internet) are more closely associated with meeting recommendations for health behaviors and cancer screening. Utilizing data from the 2005 and 2007 Health Information National Trends Surveys (HINTS), the study reveals that participants relying on print media and community organizations were more likely to meet health behavior recommendations in 2005. Conversely, in 2007, utilizing healthcare providers for health information was associated with meeting health behavior recommendations, particularly for cancer screening. These findings underscore the consistent roles of print media and interpersonal sources in influencing self-reported health behaviors, underscoring the need for further exploration of their impact on clinical outcomes and the development of social network interventions for promoting health behaviors. Various other studies are listed in Table 1.

**Table 1:** List of studies conducted all over the world on the use of print media as a tool in various health sectors

S. no.	Authors	Publishing year	Description of work/research/study	Result
1	Snyman [8]	2004	Aims to enhance the effectiveness of disseminating information on schizophrenia to South African patients and caregivers through printed brochures.	The study concludes that adherence to guidelines, coupled with audience consultation and sensitivity to social realities, can facilitate successful and cost-effective utilization of the printed medium for health information dissemination in South Africa.
2	Snyman [8]	2004	Assesses printed brochures about schizophrenia, offering a checklist and best practices for dissemination. Research reveals challenges in meeting health message criteria, emphasizing guideline adherence and social sensitivity.	Findings show brochures often lack effectiveness, with readability suggesting unsuitability for the general public.
3	Marks et al. [6]	2006	In this study, researchers aimed to compare the effectiveness of a web-based physical activity intervention with a printed workbook among adolescent girls.	Both the web-based and print-based interventions led to significant improvements in physical activity self-efficacy and intentions among adolescent girls.
4	Leask et al. [7]	2010	This study delves into the influential role of mass media in shaping health-related behaviors and perceptions, with a specific focus on how journalists in Australia select and mold news concerning avian influenza and pandemic planning.	Health and medical reporters, with their advanced technical knowledge, access to relevant sources, organizational influence, and advocacy skills, play a unique and impactful role within the print media landscape.
5	El-Jardali et al. [10]	2015	This Lebanese study investigates the role of print media in shaping health policies, examining factors influencing the quality of health reporting and the incorporation of evidence.	The study examined 1,279 health-related news articles, revealing that only 318 referenced evidence, primarily expert opinions (39.8%) over peer-reviewed research (5.9%). The quality assessment indicated low reporting standards. Interviews highlighted challenges with evidence utilization and investigative journalism.
6	Ntlotlang and Grand [11]	2016	This paper investigates the role of public libraries in disseminating health information in Botswana's Kgatleng and Kweneng districts, examining how these libraries market health information services to their communities.	The study underscores the importance of collaboration between public libraries, health agencies, and media organizations to enhance health information dissemination and user accessibility.
7	Nagler et al. [12]	2016	Analyzing local print news in two New England cities, this study explores the prevalence and framing of health disparities and social determinants of health (SDH).	The study underscores the need for targeted interventions in local media to enhance awareness of health disparities, particularly in cancer.
8	Shakeel et al. [13]	2017	Examining physicians' perceptions in Karachi, Pakistan, this cross-sectional study from January to July 2015 delves into the impact of electronic and print media on patients' health.	Most physicians doubt the media's role in lifestyle modification and question its reliability for health information, emphasizing a stronger trust in healthcare professionals.
9	Shakeel et al. [13]	2017	This study investigates physicians' perceptions regarding the impact of print and electronic media on patients' health status.	Despite the potential of mass media to raise awareness, physicians remain unconvinced about its extensive use.
10	Kim and Jung [14]	2017	Investigating the link between media use, health information-seeking behavior, and vaccination among Korean adults, this research explores the role of diverse media channels in promoting vaccination awareness.	Increased vaccination odds are linked to accessible information on diseases, radio listening, and newspaper reading.
11	Peacock et al. [15]	2019	Examining 76 newspaper/magazine articles from the UK, Romania, and Italy, this study investigates how print media depict the risks and benefits of herbal supplements.	While most articles maintained factual accuracy, they often lacked context and impartiality. The study emphasizes the need for an accessible, objective information source to empower consumers in making informed choices about herbal supplements.
12	Campbell and Rudan [16]	2020	This study addresses the crucial need for effective communication of health research to maintain public support and counteract online misinformation, particularly as new generations increasingly self-educate online.	The study explores strategies for promoting health issues online and through mass media to engage wider audiences.
13	Ramondt and Ramírez [17]	2020	This study analyzes print news coverage of air pollution health risks and precautionary measures in the USA's most polluted region over five years to assess its impact on environmental health literacy.	Overall, the findings indicate a lack of comprehensive reporting that could enhance environmental health literacy despite rising air pollution levels.
14	John and Kapilashrami [18]	2021	This study investigates the portrayal of migrants and refugees, focusing on their health, in Indian print media before the COVID-19 pandemic. Employing frame and content analyses on three English-language newspapers from January 1, 2017, to December 31, 2018, the research explores variations based on social positions.	The media's role in potentially vilifying migrants is underscored through biased framing and limited representation of migrants' voices.
15	He and Li [19]	2021	This study uses China HINTS 2017 survey data to compare traditional mass media and social media impacts on cancer information-seeking and avoidance.	A positive association was found between cancer fatalism and seeking/avoiding intentions, revealing a complex interplay of negative beliefs in information behaviors.
16	Fatimah et al. [20]	2021	Analyzing communication media and its impact on reproductive health in North Toraja society, this quantitative-descriptive study explores preferences among adolescents.	Printed communication media, particularly books and magazines, emerged as the preferred source for reproductive health information (57.39%), followed by websites (27.83%) and social media (14.78%).
17	Kersbergen et al. [5]	2022	This research explores media coverage of UK low-risk drinking guidelines from 2014 to 2017, analyzing 500 articles for reporting accuracy, tone, and purpose. Thematic analysis was applied to 200 randomly selected articles.	Media coverage peaked (7.4%) during the 2016 guidelines revision. The study emphasizes nuanced media portrayal, where neutral overall coverage coexists with critical, in-depth perspectives.

Health Education: The Role of Print Media in COVID-19 Awareness

In combating the COVID-19 pandemic, health education has emerged as a critical tool, with print media playing a pivotal role in disseminating crucial information to the general public [21]. Mass media, including newspapers and other print platforms, have been instrumental in providing timely updates and guidelines to encourage safe practices among individuals. Amid the pandemic, individuals who previously had limited engagement with news media have now incorporated it into their daily routines, underscoring the profound impact of print media on shaping public behavior [21]. Frontline workers and government agencies have intensified their efforts, spurred by the information and directives circulated through mass media channels. However, the COVID-19 crisis has brought about challenges for print media, notably a decline in newspaper supply due to concerns about potential transmission through physical newspapers [21]. This shift has prompted many to pivot toward digital media platforms for information consumption. Despite its importance, mass media has been associated with misinformation and fear during the pandemic. Studies have highlighted the influence of media exaggeration and generated fear in public perceptions, emphasizing the need for accurate and responsible reporting [21].

A study conducted in Rawalpindi, Pakistan, underscores the effectiveness of media-driven health education initiatives [22]. Through a pre-post design quantitative study, it was found that exposure to media and knowledge regarding COVID-19 increased over time. Notably, more frequent use of electronic media was associated with greater knowledge gains. Similarly, during the COVID-19 crisis, Kerala, India, excelled in crisis communication, ensuring effective management. Pre-crisis strategies prepared stakeholders, while direct engagement and media handling built trust [23]. Positive framing and stakeholder involvement further enhanced crisis response, establishing a model for future crisis communication efforts. While challenges persist, particularly regarding misinformation and media consumption habits, the role of print media in COVID-19 awareness remains indispensable. Continued efforts to promote accurate reporting and health education through print media are essential in mitigating the impact of the pandemic and fostering community resilience.

Health Promotion and Awareness

Printed educational materials (PEMs) as passive dissemination strategies in healthcare. Traditionally presented in paper formats, such as monographs and peer-reviewed journals, PEMs aim to improve clinical practice and patient outcomes [4]. Educational print materials for young breast cancer survivors (YBCS) serve as supplementary resources in patient education, yet little attention is paid to their readability and effectiveness in promoting comprehension and action [24]. This study aimed to evaluate the readability, understandability, and actionability of commonly distributed breast cancer survivorship print materials [25]. Through an environmental scan of outpatient oncology clinics in a rural Southern state, 14 materials were collected. Readability was assessed using Flesch-Kincaid, Fry Graph Readability Formula, and Simple Measure of Gobbledygook, while understandability and actionability were evaluated with the Patient Education Materials Assessment Tool for Printable Materials (PEMAT-P). Results showed that while most materials had a "difficult" readability level, they scored well in understandability and actionability [25]. This suggests that while readability is important, factors like understandability and actionability may play a more crucial role in influencing patient behavior. Personalized teaching alongside print materials could enhance comprehension and encourage action among YBCS. Despite their widespread use, there is limited understanding of how PEMs influence the dissemination of health information to patients. This underscores the need for further research to explore the impact of PEMs on patient health literacy and behavior change.

In today's information age, various forms of media, including print media, play a crucial role in shaping public perceptions and priorities. With the rise of mass media like audiovisual, print, and social media, there is a significant opportunity to leverage these platforms for health education and awareness campaigns. Studies have shown that exposure to mass media advertisements can influence public perception of health issues such as smoking and alcohol dependence. News coverage on health matters also holds considerable significance in shaping public opinion and policymaker decisions. Advertisements are particularly effective in reaching a wide audience, making print media a valuable tool for health promotion. A content analysis of health-related advertisements in Kannada newspapers revealed a focus on products and services, with fewer advertisements aimed at raising awareness [25]. While health-related product advertisements dominated, there was a notable lack of awareness-raising advertisements. These ads typically appeared on significant days like World Milk Day and World Environment Day, but their frequency could be increased to enhance effectiveness. Despite the prevalence of advertisements, studies have shown that public attention to health-related advertisements is relatively low [25]. Out of 100 outpatients surveyed, only a small percentage recalled health awareness-related advertisements, indicating a potential lack of trust and engagement with commercial advertisements [25]. To address this, there's a need for more government-sponsored advertisements clearly labeled as such to build public confidence in health-related messages.

Print media impacts on public health outcome

Health Behavior Change

The utilization of print media in mass media campaigns has emerged as a cornerstone in public health endeavors aimed at instigating behavior change on a large scale. These campaigns encompass a wide array of health-related behaviors, ranging from addressing tobacco and alcohol use to initiatives focused on heart disease prevention and cancer screening. Through channels, such as newspapers, magazines, and other print media outlets, these campaigns reach broad audiences, often in a passive manner, as part of individuals' routine media consumption [26]. Leveraging print media, mass media campaigns disseminate behaviorally focused messages repeatedly over time with the aim of invoking cognitive and emotional responses in individuals. These messages are strategically crafted to influence decision-making processes, eliminate barriers to change, and foster healthy social norms. By associating positive emotions with behavior change, these campaigns bolster intentions and enhance the likelihood of individuals adopting new behaviors [26].

Furthermore, print media campaigns exert an indirect influence on behavior change by setting agendas for interpersonal discussions and shaping social norms within communities. Changes in behavior that become norms within social networks can sway individuals' decisions, even if they were not directly exposed to campaign messages. Moreover, mass media campaigns can spark public discourse on health issues, potentially leading to changes in public policy that restrict certain behaviors. While mass media campaigns have shown promise in promoting behavior change, their effectiveness can be affected by various factors such as funding, media environment, message format, and audience characteristics. Hence, meticulous planning and evaluation are imperative to maximize the impact of print media campaigns on public health outcomes and ensure sufficient population exposure to behavior change messages.

Community Engagement

Print media campaigns have played a crucial role in influencing behaviors related to tobacco, alcohol, and illicit drug use. With tobacco being a leading cause of premature deaths worldwide, print media campaigns have been widely employed to combat tobacco use [26]. These campaigns, often integrated into broader tobacco control programs, have yielded promising outcomes in reducing smoking initiation among young people and enhancing smoking cessation rates among adults [4]. Combining mass media efforts with school and community programs has proven particularly effective in curbing smoking uptake among youth and decreasing adult smoking prevalence [27]. While evidence has predominantly been gathered from high-income countries, there's a pressing need to ensure the evidence-based implementation of such campaigns in low-income and middle-income countries, as well as among socioeconomically disadvantaged populations. Despite these successes, print media campaigns face challenges in addressing alcohol misuse due to pervasive alcohol marketing and entrenched societal norms around drinking. Efforts to promote safe drinking through print media campaigns have encountered obstacles, with messages often perceived as ambiguous by recipients. Similarly, initiatives targeting behaviors related to illicit drug use through mass media campaigns have been limited, with most research conducted in the USA. While some campaigns have demonstrated positive effects, others have been criticized for overstating their impact or inadvertently normalizing drug use.

In conclusion, print media campaigns have shown potential in influencing behaviors related to tobacco, alcohol, and illicit drug use. However, their effectiveness is influenced by various factors, including message content, audience characteristics, and concurrent exposure to other influences such as marketing tactics. Further research and investment are necessary to optimize the design and implementation of print media campaigns to achieve meaningful behavior change in these domains. On the other hand, it is worth noting that print media may not always be the most effective option, as indicated by studies examining physicians' perspectives on the impact of both electronic and print media on patient health outcomes [13]. While recognizing the potential of mass media to raise awareness among patients, a significant portion of physicians remain skeptical about the effectiveness of print media in modifying patient lifestyles or significantly influencing health outcomes. This suggests that while print media may serve as a resource for increasing patient awareness, its impact on tangible health outcomes as perceived by physicians is limited. Additionally, research by Wilson et al. suggests that while multimedia is considered promising for patient education [28], most studies found no significant difference between multimedia and print in terms of patient outcomes. This implies that print media can be equally effective as multimedia in certain contexts for disseminating patient education materials. However, the study also highlights a lack of patient involvement in material development and insufficient assessment of material readability, indicating areas for improvement in utilizing print media effectively for health outcomes.

Challenges in utilizing print media for health information

Dissemination

Medical issues frequently make headlines in print media, influencing public perception, policy decisions, and healthcare practices. While the intention is to provide accurate and informative content, medical reporting often falls short and is criticized for being speculative, inaccurate, and misleading. Understanding the challenges faced by medical journalists can offer insights into improving the quality of health information conveyed through print media [29]. A comprehensive investigation revealed several obstacles hindering the improvement of informative value in medical journalism within print media. These barriers include limitations in time, space, and knowledge, as well as competition for audience attention and editorial constraints. Additionally, difficulties arise with technical terminology, sourcing reliable information, and navigating commercial pressures. Among these challenges, time constraints and limited resources emerged as the most prevalent issues. The nature of these obstacles varies depending on the type of media outlet and the experience level of journalists. Many health reporters express challenges in finding independent experts willing to provide assistance and highlight the need for editors to undergo further education in critically evaluating medical news. Despite these hurdles, the overwhelming consensus among respondents is the importance of enhancing the informative value of medical reporting. To address these constraints, collaborative efforts between healthcare professionals and journalists are essential. Strategies to overcome these challenges may include providing journalists with access to reliable background information online, facilitating education on critical appraisal skills for editors, and fostering partnerships between journalists and experts in the medical field. Furthermore, there is a strong interest among journalists in participating in trials aimed at evaluating strategies to overcome these identified constraints. In conclusion, while medical journalists recognize the significance of delivering valid and accurate information through print media, they acknowledge the multitude of obstacles impeding their efforts. Addressing these challenges will require concerted efforts from both healthcare professionals and journalists, employing a diverse range of strategies to enhance the quality of health reporting in print media.

Health Literacy

Print media plays a crucial role in health literacy among individuals. For instance, older adults influence their understanding of health information and subsequent health outcomes [27]. Research indicates that older adults with lower health literacy tend to have lower income and education levels, rate their health as poor or fair, and experience visual or auditory difficulties. These individuals often require assistance with tasks, such as filling out forms, reading newspapers, or writing notes, highlighting the challenges they face in accessing and comprehending health information. Moreover, older adults with lower health literacy tend to use print media sources less frequently compared to those with higher health literacy levels. These findings underscore the importance of addressing health literacy disparities among older adults to improve overall health outcomes and reduce health disparities. Interventions aimed at enhancing health literacy should focus on recognizing the specific needs and limitations of older adults, ensuring that print media materials are accessible, easy to understand, and tailored to their demographic characteristics and health literacy levels. By addressing these challenges, healthcare providers and policymakers can better support older adults in accessing and utilizing print media resources to make informed decisions about their health.

Accessibility

The accessibility of print media faces significant challenges in the current digital, mobile, and social media landscape, characterized by intense competition for attention and advertising revenue [16]. As more people turn to digital platforms, particularly via mobile devices and social media, traditional print media outlets are experiencing a decline in their role as primary distributors of news. Instead, large technology companies dominate the distribution of news content and digital advertising, further marginalizing legacy media sources like newspapers and broadcasters. Legacy media organizations are under pressure to adapt to this new environment by investing in digital opportunities, cutting costs, and seeking market consolidation [21]. However, despite substantial investments in digital operations, few legacy media outlets have been able to generate profits from online news, as competition for attention and advertising remains fierce. Similarly, digital-born news organizations face challenges in monetizing their operations and competing for audience engagement in a crowded online landscape. For citizens, the shift to a digital, mobile, and social media environment offers increased choice and convenience in accessing news content. However, concerns remain about the impact of media pluralism, as a limited number of large players dominate the digital media ecosystem. While internet users have access to diverse sources and perspectives, the concentration of power among a few major platforms raises questions about media diversity and the potential for market failure in the production of independent journalism.

Print Media vs. Digital Media: Challenges to Print Media

The transition from print media to digital media presents significant challenges for traditional journalism, particularly in local contexts where journalists play a vital role in community engagement. In today's digital environment, journalists face the daunting task of redefining their relationship with audiences, utilizing digital tools to foster greater interaction and engagement [30]. A survey of 107 journalists from 42 newsrooms in the central region of Portugal revealed a widespread integration of the internet, social media, and mobile devices into journalists' workflows [31]. Digital technologies are predominantly used for news gathering and communication with sources, highlighting their importance in the journalistic process. However, the use of social media to engage directly with the community remains limited, with only a few journalists actively utilizing these platforms for community interaction. Furthermore, there is a reluctance among local journalists to recognize or incorporate content produced by citizens, indicating a potential disconnect between journalists and the communities they serve. Despite claiming to prioritize closeness to the public, many journalists may not fully embrace opportunities for community engagement offered by digital media. Overall, the findings underscore the challenges faced by print media in adapting to digital environments and leveraging digital tools to enhance community engagement. While digital technologies offer new opportunities for journalists to connect with audiences, there is a need for greater integration of social media and citizen-generated content into journalistic practices to foster meaningful engagement with local communities.

## Conclusions

Print media has historically played a vital role in public health communication, disseminating health information, and promoting healthy behaviors through newspapers, magazines, and other tangible formats. Despite challenges posed by digitalization and social media dominance, print media remains relevant, particularly during public health crises like the COVID-19 pandemic, offering extensive coverage to inform and educate the public. While print media campaigns have shown effectiveness in promoting behavior change, addressing funding constraints and message ambiguity remains a challenge. Strategic planning, evaluation, and targeted dissemination are crucial for maximizing print media's impact, but ensuring accessibility amid digital dominance requires policy reforms and efforts to enhance medical reporting and health literacy. Despite these challenges, print media's enduring importance in health communication underscores its potential to shape public perceptions and promote health education in the future.
